# Association of early glycemic change with short-term mortality in lobar and non-lobar intracerebral hemorrhage

**DOI:** 10.1038/s41598-021-95453-1

**Published:** 2021-08-09

**Authors:** Paola Forti, Fabiola Maioli, Marco Zoli

**Affiliations:** 1 Department of Medical and Surgical Sciences, University of Bologna, Via Massarenti 9, 40138 Bologna, Italy; 2 Medical Department of Integrated Care Models, Maggiore Hospital Carlo Alberto Pizzardi, Bologna, Italy

**Keywords:** Stroke, Outcomes research

## Abstract

The association between early glycemic change and short-term mortality in non-diabetic patients with acute intracerebral hemorrhage (ICH) is unclear. We retrospectively investigated non-diabetic patients with lobar (n = 262) and non-lobar ICH (n = 370). Each patient had a random serum glucose test on hospital admission and a fasting serum glucose test within the following 48 h. Hyperglycemia was defined as serum glucose ≥ 7.8 mmol/l. Four patterns were determined: no hyperglycemia (reference category), persistent hyperglycemia, delayed hyperglycemia, and decreasing hyperglycemia. Associations with 30-day mortality were estimated using Cox models adjusted for major features of ICH severity. Persistent hyperglycemia was associated with 30-day mortality in both lobar (HR 3.00; 95% CI 1.28–7.02) and non-lobar ICH (HR 4.95; 95% CI 2.20–11.09). In lobar ICH, 30-day mortality was also associated with delayed (HR 4.10; 95% CI 1.77–9.49) and decreasing hyperglycemia (HR 2.01, 95% CI 1.09–3.70). These findings were confirmed in Cox models using glycemic change (fasting minus random serum glucose) as a continuous variable. Our study shows that, in non-diabetic patients with ICH, early persistent hyperglycemia is an independent predictor of short-term mortality regardless of hematoma location. Moreover, in non-diabetic patients with lobar ICH, both a positive and a negative glycemic change are associated with short-term mortality.

## Introduction

A transitory hyperglycemia is frequent after acute stroke as a part of the acute stress reaction^1^. Admission hyperglycemia is associated with several adverse outcomes of ischemic stroke, including short-term mortality, and the association is stronger among non-diabetic than diabetic patients^2^. Studies of ischemic stroke also showed that measures of glycemic change within the first 48 h are better prognostic factors than a single glucose test on admission, since both persistent and delayed hyperglycemia are associated with poor stroke outcomes^3–5^.

Research has consistently shown that admission hyperglycemia and short-term mortality are also related in patients with primary intracerebral hemorrhage (ICH), but it is still controversial whether hyperglycemia independently contributes to hemorrhagic injury or it is just a surrogate marker for severe ICH^6^. Admission hyperglycemia is actually associated with several admission features of ICH severity such as hematoma volume and intraventricular extension^7,8^. A number of studies reported that the association with ICH mortality was independent of clinical and radiological features of ICH severity^7–14^, but most of this evidence was based on a single glucose measurement, usually the first random test on admission. Published research on the association of glycemic change with ICH mortality is very scant^14,15^.

This retrospective study investigated the association of early glycemic change with short-term mortality in non-diabetic patients with lobar and non-lobar ICH. The study also tested whether inclusion of early glycemic change would improve the prognostic ability for short-term mortality of the ICH score^16^, which is the most widely known scale for prognosis estimation in acute ICH patients^17^.

## Methods

This is a retrospective observational single-center study based on a cohort of 880 patients aged ≥ 18 year who, between January 2006 and December 2018, were consecutively admitted to the Emergency Department of the Maggiore Hospital (Bologna, Italy) within 24 h after ICH onset and subsequently transferred to the local Stroke Unit (SU) within 24 h after hospital admission. Diagnosis of ICH was based on clinical and neuroimaging data (all patients had at least one CT-head at admission). The cohort did not include patients with subarachnoid hemorrhage or intracerebral hemorrhage associated with an aneurysm, arteriovenous malformation, or other structural lesions. At SU admission, written informed consent for future research use of all data included in their medical record was sought from patients or their legally authorized representatives. The Maggiore Hospital Ethics Committee approved the study in accordance with the relevant guidelines and regulations. The data that support the findings of this study are available from the corresponding author upon reasonable request.

Demographics, preadmission medical history, and ICH characteristics were abstracted from medical records. Random serum glucose (RSG) was defined as the first available serum glucose measurement obtained after hospital admission (median time 1 h, 25th–75th percentiles, 1–2 h, range 1- 3 h). Fasting serum glucose (FSG) and glycated hemoglobin ([HbA1c], Diabetes Control and Complications Trial aligned results) were determined the morning after SU admission (median time after hospital admission 24 h; 25–75th percentile, 16–26 h; range, 12–48 h), as a part of routine biochemistry tests performed on a venous blood sample drawn after an overnight fast. All measurements were performed using automated methods at the same central laboratory. According to European stroke guideline^18^, the local protocol for management of acute ICH recommends avoidance of glucose-containing intravenous fluids and a glucose target between 7.8 and 10.0 mmol/l. Type and dose of antidiabetic treatment is at discretion of the treating physician. For the purposes of this study, only non-diabetic patients were included. Patients with diabetes were identified on the basis of pre-admission diagnosis (self-report, evidence from available medical records, or use of antidiabetic drugs; n = 136), new diagnosis reported in the SU discharge letter (n = 17), or retrospective diagnosis (n = 44). Both new and retrospective diagnosed were based on findings of admission HbA1c ≥ 48 mmol/mol (6.5%)^19^. HbA1c is not fully concordant with traditional blood glucose criteria^20^ but is a convenient choice in patients with acute stroke because, differently from blood glucose criteria, remains unaffected by stress response^21^. We also excluded patients who refused/were unable to provide informed consent (n = 20), missed laboratory data (n = 8), had unknown vital status at follow-up (n = 9), or were diagnosed with primary intraventricular hemorrhage (n = 14).

Prestroke characteristics included: disability (admission modified Rankin Scale^22^ > 1), heart disease (any history of coronary heart disease or congestive heart failure), and anticoagulant therapy. Severity of stroke on admission was measured using the National Institues of Health Stroke Scale^23^ (NIHSS) and the Glasgow Coma Scale^24^ (GCS). Hematoma location, classified as lobar versus non-lobar, hematoma volume^25^, and presence of intraventricular extension on admission were derived from the first available CT-scan as assessed by the on-duty neuroradiologist. All-cause mortality at 30 days after ICH onset was ascertained from Italian Regional Mortality Registries.

### Glycemic change

Hyperglycemia in non-diabetic patients was defined as serum glucose ≥ 7.8 mmol/L based on the institutional threshold for treatment. Using RSG and FSG, we defined four patterns of glycemic change: no hyperglycemia (both RSG and FSG below 7.8 mmol/L); persistent hyperglycemia (high RSG and high FSG), delayed hyperglycemia (high FBG only), and decreasing hyperglycemia (high RSG only). Hypoglycemia (< 3.9 mmol/l^26^) was too rare for meaningful analyses (< 0.1% of all RSG values and only 5% for FSG). Dichotomization of continuous predictors can lead to loss of information, reduction in statistical power, and poor fit of the association of interest^27^. Therefore, we also calculated continuous glycemic change (FSG minus RSG) as an alternative to the categorical indicator: a positive change suggests an increasing glycemic pattern whereas a negative change suggests a decreasing pattern.

### Statistical analysis

Variables were presented as median (25th–75th percentile) or number (percentage). Univariate associations were tested using Kruskall-Wallis test or chi-square test as appropriate. The multivariable-adjusted association of glycemic change categories with 30-day mortality was tested using adjusted Hazard Ratios (HR) and their 95% confidence intervals (95%CI) from Cox regression models also including age, sex, disability, heart disease, anticoagulant use, GCS, hematoma volume, and prevalence of intraventricular hematoma extension. NIHSS was not included as a covariate because of its high collinearity with GCS, which is the usual instrument of choice for initial assessment of ICH severity^28^. Cox models for glycemic change as a continuous variable also included admission RSG. To allow for nonlinear associations, continuous glucose measurements were modelled using restricted cubic splines (RCS) with 4 knots placed at pre-specified locations (5th, 35th, 65th, and 95th percentile) of the variable distribution^27,29^. Non-linearity was rejected for a conservative P-value > 0.100^29^. HRs were calculated only within the range of actually observed values^30^. The reference pattern for glycemic change categories no hyperglycemia. The reference value for continuous glycemic change was set at the minimum mortality point. The reference value for RSG was set at 5.5 mmol (upper range of normal blood glucose^19^). C-statistic was used to compare the prognostic value of Cox models using only the ICH score (calculated from the study covariates GCS, hematoma volume, presence of intraventricular hematoma, infratentorial origin, and age^16^) and models including the ICH score along with the study measures of glycemic change. Analyses were performed with R software version 3.5.3 and Harrell’s rms package^31^. Significance for P-value was set at 0.050 (two-tailed). For both the lobar and non-lobar subgroup, statistical power was 0.80 for a HR of 2.0.

## Results

The final cohort included 632 patients (age range, 34 to 104 years). Preliminary multivariable-adjusted Cox models for mortality prediction in the study cohort considered as whole confirmed a significant interaction of hematoma location with glycemic change (p < 0.001, both categorical and continuous).

Table 1 summarizes patient characteristics for lobar and non-lobar ICH. Lobar ICH was associated with higher hematoma volume and lower prevalence of intraventricular hematoma extension than non-lobar ICH, whereas clinical severity did not differ by location. The most frequent category of glycemic change in both lobar and non-lobar ICH was no hyperglycemia, followed by decreasing hyperglycemia. However, lobar ICH had a borderline higher occurrence of persistent and delayed hyperglycemia than non-lobar ICH. Glycemic change as a continuous variable was also smaller in lobar than in non-lobar ICH. Mortality rate did not differ by hematoma location.

**Table 1:** Characteristics of the study cohort according to location of intracerebral hemorrhage.

Variable	Lobar (n = 262)	Non-lobar (n = 370)	*P* value
Age, years	79 (72–84)	77 (67–84)	0.063
Male sex	115 (43.9)	181 (48.9)	0.212
Prestroke disability	93 (35.5)	97 (26.2)	0.012
Heart disease	36 (13.7)	45 (12.2)	0.559
Anticoagulant use	38 (14.5)	48 (13.0)	0.580
National Institutes of Health Stroke Scale	11 (4–21)	10 (4–19)	0.565
Glasgow Coma Scale	14 (10–15)	14 (10–15)	0.483
Volume, cm^3^	29 (8–82)	7 (2–19)	< 0.001
Intraventricular hemorrhage	78 (29.8)	138 (37.3)	0.049
Random serum glucose, mmol/l	6.8 (5.7–8.9)	6.7 (5.8–7.9)	0.830
Fasting serum glucose, mmol/l	5.5 (4.8–6.6)	5.2 (4.6–6.1)	0.003
**Glycemic change, categorical**			0.070
No hyperglycemia	175 (66.8)	259 (70.0)	
Persistent hyperglycemia	16 (6.1)	12 (3.2)	
Delayed hyperglycemia	14 (5.3)	9 (2.4)	
Decreasing hyperglycemia	57 (21.8)	90 (24.3)	
Glycemic change, mmol/l	-0.9 (-2.1 to -0.1)	-1.3 (-2.3 to -0.4 )	0.005
Glycated haemoglobin, mmol/mol	38 (34–41)	38 (34–41)	0.957
Death at 30 days	68 (25.9)	87 (23.5)	0.543

Data are median (25th–75th percentile) or n (%). To convert mmol/l to mg/dl, multiply by 18.018.

Table 2 summarizes multivariable-adjusted analyses for categories of glycemic change by hematoma location. Persistent hyperglycemia was associated with higher mortality in both lobar and non-lobar ICH. Delayed and decreasing hyperglycemia were associated with higher mortality only in lobar ICH.

**Table 2:** Association of glycemic change categories with 30-day mortality according to hematoma location.

Mortality	No hyperglycemia	Persistent hyperglycemia	Delayed hyperglycemia	Decreasing hyperglycemia	P-value
**Lobar**					
N all	175	16	14	57	
n (%) cases	25 (14.3)	10 (62.5)	10 (71.4)	23 (40.3)	< 0.001*
HR (95%CI)	1.00	3.00 (1.28–7.02)	4.10 (1.77–9.49)	2.01 (1.09–3.70)	0.003 †
**Non-Lobar**					
N all	258	12	9	90	
n (%) cases	46 (17.8)	11 (91.7)	2 (22.9)	28 (31.1)	< 0.001*
HR (95%CI)	1.00	4.95 (2.20–11.09)	0.77 (0.18–3.34)	1.44 (0.88–2.37)	0.001†

*P-value from Chi-square.

^†^P-value for overall association from a Cox model adjusted for age, sex, prestroke disability, heart disease, anticoagulant use, Glasgow Coma Scale, hematoma volume and intraventricular hemorrhage.

**Figure 1:**
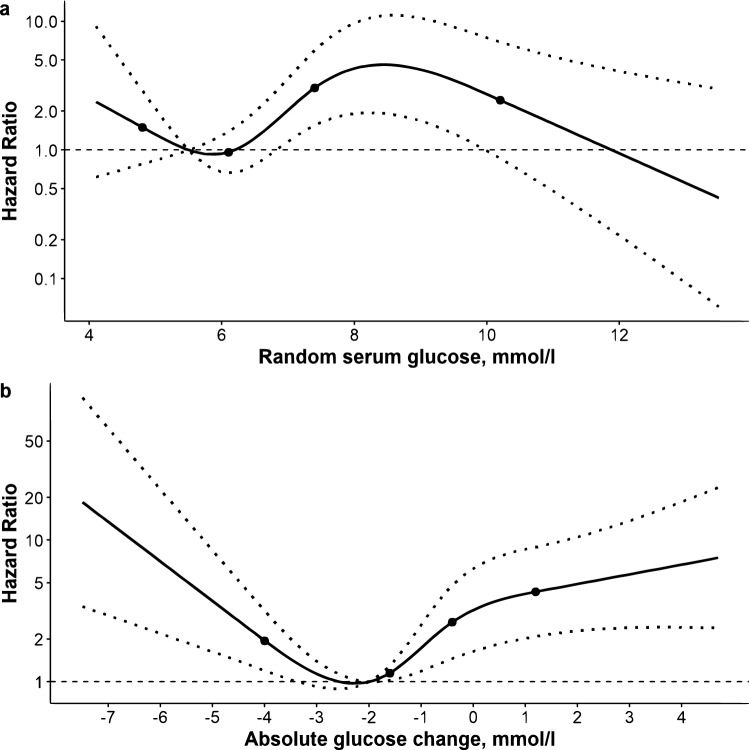
Association of admission random serum glucose and glycemic change with 30-day mortality after lobar intracerebral hemorrhage. Panel (**a**) refers to random serum glucose (RSG) on admission and Panel (**b**) to glycemic change within the following 48 h in 245 non-diabetic patients. Hazard ratios (solid line) and 95% confidence intervals (dotted lines) are from a Cox regression model using restricted cubic splines with four knots (represented by dots) located at the 5th, 35th, 65th, and 95th percentiles of each glucose measure. Reference value is set at 5.5 mol/l for RSG and at the minimum mortality point for glycemic change. Models were adjusted for age, sex, prestroke disability, heart disease, anticoagulant use, Glasgow Coma Scale, hematoma volume and prevalence of intraventricular hemorrhage. To convert mmol/l to mg/dl, multiply by 18.018.

Multivariable-adjusted models using admission RSG and glycemic change as continuous variables showed that both predictors were associated with mortality. In lobar ICH, however, there was evidence of a significant non-linear component (overall and non-linear p < 0.001 for both RSG and glycemic change). According to the RCS model, the HR for mortality associated with admission RSG (Fig. 1a) significantly increased only for values between 7 and 10 mmol/L, whereas mortality and glycemic change had a clear U-shaped association (Fig. 1b), with a nadir of risk at around − 2.

Multivariable-adjusted models for non-lobar ICH showed that mortality was associated with both RSG (overall p < 0.001) and glycemic change (overall p = 0.007) but RCS modeling was unnecessary, because there was no evidence of non-linearity (non-linearity p > 0.200 for both predictors). Final estimates for multivariable-adjusted HR from a standard Cox model were 1.29 (95% CI 1.11–1.49) for each 1 mmol/l increase in RSG and 1.37 (95% CI 1.14–1.64) for each + 1 mmol/l increase in absolute glycemic change.

In analyses pooling toghether lobar and non-lobar ICH, C-statistic for mortality prediction of the model including only the ICH score was 0.748 (95% CI 0.703–0.793). Overlapping values of C-statistic were found for the models including ICH score along with categories of glycemic change (0.769, 95% CI 0.726–0.812) or rSG and glucose change as continuous predictors (0.798, 95% CI 0.761–0.835).

## Discussion

This retrospective study shows that early glycemic change is associated with short-term ICH mortality independent of major clinical and neuroradiological features of ICH severity. However, patterns of associations differ by hematoma location. We chose a priori to perform separate analyses for lobar and non-lobar ICH because of their different risk factors, presentation characteristics, and outcome^32,33^. This choice was supported by preliminary analyses of the whole study cohort showing a significant interaction of glycemic change with hematoma location.

A first set of analyses used four patterns of glycemic change, derived from the dichotomization of admission RSG and the first available FSG. In lobar ICH, short-term mortality was associated with hyperglycemia independent of whether the pattern was persistent, delayed or decreasing. By contrast, mortality after non-lobar ICH was associated only with persistent hyperglycemia.

A second set of analyses simultaneously testing admission RSG and glycemic change as continuous predictors confirmed that they both have independent associations with mortality. According to our model, the increase in mortality risk for increasing values of admission RSG was non-linear for lobar ICH and linear for non-lobar ICH. However, the apparent downward turn of HR estimates for RSG above 10 mmol/l in lobar ICH must be taken with caution because prediction from RCS models is less reliable at the tails of a variable distribution^27^. Our findings agree with previous evidence that admission RSG is an independent predictor of short-term ICH mortality^7–14^. The association was not confirmed in two cohorts including a large proportion of patients with very severe ICH^34,35^. However, in very severe ICH, major predictors of short-term mortality such as clinical and neuroradiological features are known to obscure the role of weaker predictors such as admission hyperglycemia^11^.

Our second set of analyses also showed that, independent of admission RSG, mortality risk additionally varied with glycemic change. In lobar ICH the association was clearly U-shaped, with mortality increasing for both positive and negative glucose variations from a nadir around − 2 mmol/L. This negative nadir reflects the spontaneous decline in glycemia that occurs in the majority of patients during the early phase of an acute stroke^36^. By contrast, the association of glycemic change with mortality after non-lobar ICH was linear and only a positive glycemic change was associated with an increased mortality risk.

It is still debated whether stress hyperglycemia is a true effector of secondary brain damage after ICH^6^. However, animal models document an effect of hyperglycemia on early hematoma expansion by exacerbation of perihematomal neuronal death, brain edema, and blood–brain barrier disruption^37–39^. Possible physiopathological mechanisms of damage include increased oxidative stress^40^, downregulation of aquaporin-4^41^, depression of perihematomal autophagy^38^, and promotion of interleukin-1 mediated inflammatory processes^42,43^.

Existing literature also suggests some mechanisms that may underly the unfavorable association of negative glycemic change with mortality after lobar ICH. Stress hyperglycemia is an adaptive response aiming to provide the brain with ready fuel^44^. Therefore, an early fall in blood glucose may hasten energy failure in the damaged brain. Quenching of reactive oxygen species in damaged brain tissue is also dependent on glucose availability^45^. Finally, endothelial damage by oxidative stress has been found to be greater for intermittent as opposed to sustained hyperglycemia^46,47^.

The lack of an adverse mortality effect for decreasing glycemic change in non-lobar ICH might be explained by a lower local vulnerability to secondary ischemic damage. Impaired vasoregulation due to acute hyperglycemia may enhance perihematomal hypoperfusion in ICH patients, so favouring the development of secondary satellite ischemia brain lesions adding to hemorrhagic damage^48,49^. As diabetes mellitus is a specific risk factor for non-lobar ICH^50^, non-diabetic patients with non-lobar ICH might have a higher likelihood of chronical exposure to some degree of unrecognized dysglycemia. Therefore, these patients might also be less vulnerable to the adverse effects of sudden glycemic drops in ischemic perihematomal areas as a consequence of having acquired a higher tolerance to dysglycemia^51^. Another possible explanation might be that only persistent hyperglycemia favours damage from hematoma hexpansion, which is usually more prononunced for non-lobar than lobar ICH^52,53^.

Existing literature about the association of post-ICH glycemic patterns and early mortality is scant. In a multicentric study of 295 patients^15^, unadjusted rate of 30-day mortality was 79.5% for persistent hyperglycemia, 40% for an increasing trajectory, 36.5% for a decreasing trajectory, and only 8.6% for normoglycemia. However, location-specific data and multivariable-adjusted estimations were not provided. In a multivariable-adjusted Finnish study of 576 patients also using four glycemic trajectories, only persistent hyperglycemia was an independent predictor of 6-month ICH mortality^14^. However, underestimation of existing associations with delayed and decreasing hyperglycemia patterns cannot be excluded, because the Finnish study had a longer follow-up and may have included patients with unrecognized diabetes, as diagnosis was based only on preadmission information. Moreover, the Finnish study dichotomized hematoma location as infratentorial versus supratentorial, but supratentorial hemorrhages are not at all homogeneous. Lobar ICH is more often associated with amyloid angiopathy whereas non-lobar ICH is more related to diabetes and hypertensive vasculopathy^33,50,54^. Moreover, although lobar ICH is characterized by a larger admission hematoma volume and worse neurological impairment, its prognosis is usually better than that of non-lobar ICH^33,54^.

Studies of patients with critical illness^51^ and subarachnoid hemorrhage^55^ support the hypothesis that glucose fluctuations may be as important as a persistent hyperglycemia for prediction of early mortality. A study of ICH patients actually found no association of glucose fluctuations with death or dependency at 3 months^56^. However, sample size was small and analyses were not stratified by hematoma location.

The major strengths of this study include: a sample size at least comparable to that of the other two available studies investigating post-ICH glycemic patterns and mortality^14,15^; the effort to identify unknown diabetes cases; and the investigation of post-ICH glycemic change using both categorical and continuous indicators. Possible bias from withdrawal of care was also small because current Italian laws do not allow formal do-not-resuscitate orders nor withdrawal of basic life sustaining treatments (hydration and nutrition). Finally, the main positive findings would remain statistically significant even using a very conservative p-values (up to < 0.001) in order to account for multiple testing.

Our study, however, has also many limitations. The first and major limitation is the size of the study subgroups, with an optimal 0.80 statistical power reached only for a HR as high as 2.0. Therefore, it cannot be excluded that insufficient statistical power is the only reason for failing to identify an association of negative glicemic change with mortality in non-lobar ICH. However, we believe this is unlikely, because the association was actually detected in the smaller of the two study subgroups. The small number of cases also limited the number of covariates to be included in the models and lead to wide 95% confidence intervals. However, our chosen covariates included all the major mortality predictors listed in the ICH score. Second, the design is retrospective and data were collected at a single hospital center. Third, glycemic change was calculated using only two measurements. Fourth, we lack data about hematoma growth and non-mortality outcomes such as post-ICH functional status. Fifth, our measures of glycemic change are unlikely to be of use in clinical practice, because adding these measures to a widely known prognostic tool for short-term mortality such as the ICH score did not significantly increase its performance. Similar findings were reported by a study evaluating whether addition of random admission hyperglycemia improved the performance of a prognostic score for short-term mortality in ischemic stroke^57^. All the same, we believe that our findings can be of interest because they suggest that, depending on hematoma location, positive and negative glucose variation may have different associations with mortality. Confirmation and further understanding of these findings on larger study cohorts might contribute to explain why previous clinical trials failed to identify clinical benefits from intensive glucose control after acute stroke^58,59^.

## References

[1] Chen R, Ovbiagele B, Feng W (2016). Diabetes and stroke: Epidemiology, pathophysiology, pharmaceuticals and outcomes. Am. J. Med. Sci..

[2] Lau L-H, Lew J, Borschmann K, Thijs V, Ekinci EI (2019). Prevalence of diabetes and its effects on stroke outcomes: A meta-analysis and literature review. J. Diabetes Investig..

[3] Fuentes B (2009). The prognostic value of capillary glucose levels in acute stroke: The GLycemia in Acute Stroke (GLIAS) study. Stroke.

[4] Muir KW, McCormick M, Baird T, Ali M (2011). Prevalence, predictors and prognosis of post-stroke hyperglycaemia in acute stroke trials: Individual patient data pooled analysis from the Virtual International Stroke Trials Archive (VISTA). Cerebrovasc. Dis. Extra.

[5] Yong M, Kaste M (2008). Dynamic of hyperglycemia as a predictor of stroke outcome in the ECASS-II trial. Stroke.

[6] Guo X (2016). Hyperglycemia and mortality risk in patients with primary intracerebral hemorrhage: A meta-analysis. Mol. Neurobiol..

[7] Appelboom G (2011). Severity of intraventricular extension correlates with level of admission glucose after intracerebral hemorrhage. Stroke.

[8] Saxena A (2016). Prognostic significance of hyperglycemia in acute intracerebral hemorrhage: The INTERACT2 study. Stroke.

[9] Fogelholm R, Murros K, Rissanen A, Avikainen S (2005). Admission blood glucose and short term survival in primary intracerebral haemorrhage: A population based study. J. Neurol. Neurosurg. Psychiatry.

[10] Kang K (2019). Association of pre- and post-stroke glycemic status with clinical outcome in spontaneous intracerebral hemorrhage. Sci. Rep..

[11] Passero S, Ciacci G, Ulivelli M (2003). The influence of diabetes and hyperglycemia on clinical course after intracerebral hemorrhage. Neurology.

[12] Stead LG (2010). Emergency Department hyperglycemia as a predictor of early mortality and worse functional outcome after intracerebral hemorrhage. Neurocrit. Care.

[13] Sun S (2016). Prognostic value of admission blood glucose in diabetic and non-diabetic patients with intracerebral hemorrhage. Sci. Rep..

[14] Wu TY (2017). Persistent hyperglycemia is associated with increased mortality after intracerebral hemorrhage. J. Am. Heart Assoc..

[15] Godoy DA, Piñero GR, Svampa S, Papa F, Di Napoli M (2008). Hyperglycemia and short-term outcome in patients with spontaneous intracerebral hemorrhage. Neurocrit. Care.

[16] Hemphill JC, Bonovich DC, Besmertis L, Manley GT, Johnston SC (2001). The ICH score: A simple, reliable grading scale for intracerebral hemorrhage. Stroke J. Cereb. Circ..

[17] Pinho J, Costa AS, Araújo JM, Amorim JM, Ferreira C (2019). Intracerebral hemorrhage outcome: A comprehensive update. J. Neurol. Sci..

[18] Fuentes B (2018). European Stroke Organisation (ESO) guidelines on glycaemia management in acute stroke. Eur. Stroke J..

[19] American Diabetes Association (2018). Classification and diagnosis of diabetes: Standards of medical care in diabetes 2018. Diabetes Care.

[20] Lipska KJ (2010). Identifying dysglycemic states in older adults: Implications of the emerging use of hemoglobin A1c. J. Clin. Endocrinol. Metab..

[21] Kernan WN (2013). Screening for diabetes after stroke and transient ischemic attack. Cerebrovasc. Dis..

[22] van Swieten JC, Koudstaal PJ, Visser MC, Schouten HJ, van Gijn J (1988). Interobserver agreement for the assessment of handicap in stroke patients. Stroke.

[23] Lyden P (2017). Using the National Institutes of Health Stroke Scale: A cautionary tale. Stroke.

[24] Teasdale G, Jennett B (1974). Assessment of coma and impaired consciousness. A practical scale. Lancet.

[25] Kothari RU (1996). The ABCs of measuring intracerebral hemorrhage volumes. Stroke.

[26] Workgroup on Hypoglycemia, American Diabetes Association (2005). Defining and reporting hypoglycemia in diabetes: A report from the American Diabetes Association Workgroup on Hypoglycemia. Diabetes Care.

[27] Desquilbet L, Mariotti F (2010). Dose-response analyses using restricted cubic spline functions in public health research. Stat. Med..

[28] Hemphill JC (2015). Guidelines for the management of spontaneous intracerebral haemorrhage. A guideline for healthcare professionals from the American Heart Association/American Stroke Association. Stroke.

[29] Harrell FE (2001). Regression Modeling Strategies: With Applications to Linear Models, Logistic Regression, and Survival Analysis.

[30] Altman DG, Bland JM (1998). Generalisation and extrapolation. BMJ.

[31] Harrell FE (2019). Regression Modeling Strategies.

[32] Gross BA, Jankowitz BT, Friedlander RM (2019). Cerebral intraparenchymal hemorrhage: A review. JAMA.

[33] Samarasekera N (2015). Influence of intracerebral hemorrhage location on incidence, characteristics, and outcome. Stroke.

[34] Bender M, Naumann T, Uhl E, Stein M (2020). Early serum biomarkers for intensive care unit treatment within the first 24 hours in patients with intracerebral hemorrhage. J. Neurol. Surg..

[35] Kongwad LI, Hegde A, Menon G, Nair R (2018). Influence of admission blood glucose in predicting outcome in patients with spontaneous intracerebral hematoma. Front. Neurol..

[36] Gray CS, Hildreth AJ, Alberti GKMM, O’Connell JE (2004). Poststroke hyperglycemia. Stroke.

[37] Chiu C-D (2012). Investigation of the effect of hyperglycemia on intracerebral hemorrhage by proteomic approaches. Proteomics.

[38] Liu R-Y, Wang J-J, Qiu X, Wu J-M (2014). Acute hyperglycemia together with hematoma of high-glucose blood exacerbates neurological injury in a rat model of intracerebral hemorrhage. Neurosci. Bull..

[39] Song E-C (2003). Hyperglycemia exacerbates brain edema and perihematomal cell death after intracerebral hemorrhage. Stroke.

[40] Won SJ, Tang XN, Suh SW, Yenari MA, Swanson RA (2011). Hyperglycemia promotes tissue plasminogen activator-induced hemorrhage by Increasing superoxide production. Ann. Neurol..

[41] Chiu C-D (2013). Hyperglycemia exacerbates intracerebral hemorrhage via the downregulation of aquaporin-4: Temporal assessment with magnetic resonance imaging. Stroke.

[42] Dror E (2017). Postprandial macrophage-derived IL-1β stimulates insulin, and both synergistically promote glucose disposal and inflammation. Nat. Immunol..

[43] Sobowale OA (2016). Interleukin-1 in stroke. Stroke.

[44] Marik PE, Bellomo R (2013). Stress hyperglycemia: An essential survival response!. Crit. Care.

[45] Robbins NM, Swanson RA (2014). Opposing effects of glucose on stroke and reperfusion injury: Acidosis, oxidative stress, and energy metabolism. Stroke.

[46] Ceriello A (2008). Oscillating glucose is more deleterious to endothelial function and oxidative stress than mean glucose in normal and type 2 diabetic patients. Diabetes.

[47] Monnier L (2006). Activation of oxidative stress by acute glucose fluctuations compared with sustained chronic hyperglycemia in patients with type 2 diabetes. JAMA.

[48] Ye X (2020). Stress-induced hyperglycemia and remote diffusion-weighted imaging lesions in primary intracerebral hemorrhage. Neurocrit. Care.

[49] Prabhakaran S, Naidech A (2012). Ischemic brain injury after intracerebral hemorrhage. Stroke.

[50] Jolink WMT (2020). Location-specific risk factors for intracerebral hemorrhage: Systematic review and meta-analysis. Neurology.

[51] Krinsley JS, Meyfroidt G, van den Berghe G, Egi M, Bellomo R (2012). The impact of premorbid diabetic status on the relationship between the three domains of glycemic control and mortality in critically ill patients. Curr. Opin. Clin. Nutr. Metab. Care.

[52] Grunwald Z (2017). Perihematomal edema expansion rates and patient outcomes in deep and lobar intracerebral hemorrhage. Neurocrit. Care.

[53] Roh D (2019). Hematoma expansion differences in lobar and deep primary intracerebral hemorrhage. Neurocrit. Care.

[54] Godoy DA, Piñero GR, Koller P, Masotti L, Di Napoli M (2015). Steps to consider in the approach and management of critically ill patient with spontaneous intracerebral hemorrhage. World J. Crit. Care Med..

[55] Kurtz P (2014). Systemic glucose variability predicts cerebral metabolic distress and mortality after subarachnoid hemorrhage: A retrospective observational study. Crit. Care.

[56] Wada S (2018). Outcome prediction in acute stroke patients by continuous glucose monitoring. J. Am. Heart Assoc..

[57] McCall SJ (2018). Hyperglycaemia and the SOAR stroke score in predicting mortality. Diab. Vasc. Dis. Res..

[58] Piironen K, Putaala J, Rosso C, Samson Y (2012). Glucose and acute stroke. Stroke.

[59] Zheng D, Zhao X (2020). Intensive versus standard glucose control in patients with Ischemic stroke: A meta-analysis of randomized controlled trials. World Neurosurg..

